# Direct and indirect assessment of perfectionism in patients with depression and obsessive-compulsive disorder

**DOI:** 10.1371/journal.pone.0270184

**Published:** 2022-10-13

**Authors:** Barbara Cludius, Sarah Landmann, Anne-Katrin Külz, Keisuke Takano, Steffen Moritz, Lena Jelinek

**Affiliations:** 1 Department of Psychiatry and Psychotherapy, University Medical Center Hamburg-Eppendorf, Hamburg, Germany; 2 Division of Clinical Psychology and Psychological Treatment, Department of Psychology, LMU Munich, Munich, Germany; 3 Clinic of Psychiatry and Psychotherapy, University of Freiburg, Freiburg, Germany; Tel Aviv University, ISRAEL

## Abstract

According to the transdiagnostic perspective, psychological disorders share common cognitive processes involved in their pathogenesis. One dysfunctional belief that has been found to be associated with several psychological disorders, including major depressive disorder (MDD) and obsessive-compulsive disorder (OCD), is perfectionism. Perfectionism comprises two factors, namely, perfectionistic strivings and perfectionistic concerns. This study aims to replicate and extend previous research in several ways. We aimed to assess similarities between the two disorders using Bayesian statistics. Furthermore, as dysfunctional beliefs are assumed to not be fully accessible by introspection, we included an indirect measure (perfectionism single category implicit association task; SC-IAT). The SC-IAT and a self-report measure of perfectionism (FMPS) was used in patients with MDD (*n* = 55), OCD (*n* = 55), and in healthy controls (*n* = 64). In replication of previous findings, patients with MDD and OCD differed from healthy controls regarding self-reported perfectionism scores. Furthermore, Bayesian statistics showed that the two patient groups did not differ regarding perfectionistic strivings and only showed differences on perfectionistic concerns, when the doubts about actions subscale–which is also closely related to symptoms of OCD–was included. Contrary to our expectations, the SC-IAT did not discriminate groups. In conclusion, these results give further evidence that self-reported perfectionism may serve as a relevant transdiagnostic process. More studies are needed to assess implicit facets of perfectionism.

## 1. Introduction

Major depressive disorder (MDD) and obsessive-compulsive disorder (OCD) are common psychological disorders with a life-time prevalence of 16.6% and 2.3%, respectively [1]. The two disorders show high life-time comorbidity rates. About 60% of patients with OCD also meet the criteria for a diagnosis of MDD [2, 3]. The transdiagnostic perspective can be used to explain those high comorbidity rates. A transdiagnostic perspective, as an alternative to disorder-specific approaches has become popular in the last years [4]. Several conceptualizations exist as to what can be defined as a transdiagnostic process relevant for mental disorders (e.g., Research Domain Criteria, RDOC [5]). One perspective was presented by Harvey et al. [4], who argue that a process should meet two criteria to be identified as a transdiagnostic. First, a transdiagnostic process must be present across at least four mental disorders. Thus, existing diagnostic categories are used as a starting point for the identification and evaluation of processes as transdiagnostic. Second, the process must be causally involved in the development and/or maintenance of a mental disorder, instead of only representing a mere epiphenomenon. Therefore, according to the transdiagnostic perspective the high comorbidity rates between MDD and OCD may indicate that the two disorders share common risk or maintaining factors such as behavioral and cognitive processes [4].

An overlap regarding vulnerabilities of the two disorders can be found when comparing cognitive models of MDD and OCD [6–8]. According to these models, dysfunctional beliefs represent vulnerability factors for the onset and the exacerbation of both disorders. One of the dysfunctional beliefs that is assumed to be apparent in both disorders is perfectionism [9, 10]. According to Stoeber and Otto [11], perfectionism is a multidimensional construct that is comprised of two factors, perfectionistic strivings and perfectionistic concerns. The perfectionistic-strivings factor is related to high standards for oneself in order to strive for perfectionism(for a theoretical article on the difference between pursuit of excellence and pursuit of perfectionism see [12]), whereas the perfectionistic-concerns factor is related to one’s self-esteem based on how well one’s own standards are met [13].

In a meta-analysis on studies using self-report measures, both factors of perfectionism consistently explained significant amounts of variance in MDD and OCD [14]. Furthermore, results from several studies suggest that perfectionism may serve as a risk factor or maintaining factor in MDD (for a recent meta-analysis see [15]). Similarly, results from treatment studies showed that perfectionism/intolerance of uncertainty was a unique predictor for OCD symptoms at post-treatment [16] and changes in perfectionism/intolerance of uncertainty preceded changes in OCD symptoms [17]. In a recent prospective study in patients with eating disorders, perfectionism positively predicted symptoms of OCD six months later [18]. Only a minority of studies assessed both MDD and OCD simultaneously. Patients with MDD and OCD reported elevated scores regarding the perfectionistic-concerns factor, but not the perfectionistic-strivings factor [19]. Patients with MDD and OCD scored higher on an item testing perfectionism compared to healthy controls [20]. In adolescents with OCD, perfectionism predicted both depressive symptoms as well as OCD symptoms [21] and perfectionism predicted treatment outcome in MDD and OCD even after controlling for pre-treatment symptoms [16]. In summary, theoretical foundations [6–8] are supported by empirical evidence using established self-report measures (e.g., [22]) that perfectionism is associated with both MDD and OCD [14]. However, to date it remains unclear whether both disorders are similarly related to the two factors of perfectionism or whether one disorder is more strongly related to perfectionism than the other [14].

Furthermore, according to dual-process models of cognition (for an overview see [23]) it can be assumed that not all relevant aspects of perfectionism can be reported in self-report measures as they may not be available to consciousness [24]. Dual-process models of cognition assume two modes of information processing [23] an associative system, which is thought to operate unintentionally or automatically and a reflective system, which is assumed to require cognitive effort and can adjust information processing in the association system. According to a dual-process model in MDD [25] dysphoric mood states develop due to a negatively biased self-referent associative system and an attenuated reflective system. It is assumed that this interplay between the two systems will lead to a downward spiral which will maintain dysphoric mood states. Beck [6] proposes that the associative system includes dysfunctional beliefs which enhance depressive symptoms once activated. The models could also be applied to OCD in that dysfunctional beliefs are assumed to influence the appraisal of thoughts in the associative system, which then increases the occurrence of intrusive thoughts as well as neutralizing efforts (for an overview see [7]). To more fully capture the dysfunctional belief of perfectionism, indirect measures should be used to assess processes in the associative system as a complement to self-report measures.

With indirect measures, dysfunctional beliefs are derived from seemingly unrelated responses to stimuli [26]. One study has used an indirect measure, the dot-probe task, to assess attentional biases to negatively valenced perfectionistic words [27]. However, no psychometric properties of the task were reported in the study and the dot-probe task has been criticized for its low reliability scores [28]. The Implicit Association Test (IAT; [29]) has shown superior psychometric properties compared to other indirect paradigms [30]. A Single Category IAT (SC-IAT; [31]) was developed by De Cuyper and colleagues [32], which evaluates how strong the participants’ associations with themselves and perfectionistic strivings is, in comparison to others. In previous studies using the perfectionism SC-IAT, assessing its psychometric properties in college students, the reliability and the validity of the task was assessed [32]. To the best of our knowledge, no reliable and valid indirect measure exists to assess the perfectionistic-concerns factor of perfectionism.

The aim of the present study was to replicate and extend previous findings on perfectionism as a possible transdiagnostic process. Using self-report measures we hypothesized higher perfectionism scores regarding both the perfectionistic-strivings and perfectionistic-concerns factor for patients with MDD and OCD compared to healthy controls. As an extension of previous findings, we expected no differences between patients with MDD and OCD regarding either factor of perfectionism when using self-report measures. Furthermore, based on dual-process models [23, 25] further extended previous research by complementing self-report measures with an established indirect measure (the perfectionism SC-IAT). We hypothesized higher scores in the perfectionistic-strivings factor in both MDD and OCD compared to a healthy control group but no differences between the two patient groups.

## 2. Methods

### 2.1 Participants

Fifty-five patients with OCD and 55 patients with MDD were recruited in the context of two larger clinical trials on MDD (study registration #DRKS00010543) and OCD (study registration #DRKS00004525). Patients with MDD were inpatients on a ward for affective disorders at a psychiatric clinic. The study was approved by the Ethics Committee of the Medical Board Hamburg (Germany). Patients with OCD were recruited through OCD and anxiety wards of psychiatric clinics, psychotherapists seeing patients on an outpatient basis, disorder specific online fora, and newspaper advertisements. The study was approved by the Ethics Committee of the Freiburg University Medical Center (Germany). Participants were excluded if they were younger than 18 or older than 70 years, had been diagnosed with any severe neurological disorder (e.g., stroke, epilepsy), mania, psychotic disorder, current substance or alcohol dependence, or mental retardation (IQ < 70). Sixty-four participants served as healthy controls and were comparable as to age, gender, and education relative to the MDD and OCD sample (see Table 1). Additional exclusion criteria for healthy controls were a lifetime diagnosis of MDD or OCD as well as any current psychiatric diagnosis, which was verified with the M.I.N.I. (see below).

**Table 1:** Demographic and psychopathological data: Mean (standard deviation).

	MDD patients (*n* = 55)	OCD patients (*n* = 55)	Healthy controls (*n* = 64)	Statistics
*Demographic characteristics*				
Age in years	42.42 (10.15)	38.73 (11.26)	39.10 (14.72)	*F*(2,171) = 1.52, *p* = .22
Sex (male/female)	28 /27	19 / 36	27 / 37	χ^2^(2) = 3.02, *p* = .22
Verbal intelligence (WST)	105.69 (9.80) ^1^	104.40 (10.03)	108.34 (9.04)	*F*(2,161) = 2.62, *p* = .08
*Psychopathology*				
BDI-II total	28.81 (13.00)	19.31 (10.49)^1^	4.20 (5.01)	*F*(2,169) = 94.84, *p* < .001
OCI-R total	-	25.49 (11.41)^2^	6.98 (6.75)	*t*(115) = 10.88, *p* < .001
Comorbid disorder (total)	32	29	-	χ^2^(1) = 0.33, *p* = .70
MDD	-	15	-	-
Dysthymia	-	12	-	-
OCD	7	-	-	-
Panic disorder	2	1	-	χ^2^(1) = 0.64, *p* = .64
Agoraphobia	9	7	-	χ^2^(1) = 0.29, *p* = .39
Social anxiety disorder	7	7	-	χ^2^(1) = 0.12, *p* = .47
Posttraumatic stress disorder	2	1	-	χ^2^(1) = 2.76, *p* = .10
Generalized anxiety disorder	12	6	-	χ^2^(1) = 2.37, *p* = .09
Alcohol abuse	5	-	-	
Psychotropic medication	44	35	-	χ^2^(1) = 3.64, *p* = .06

*Note*. MDD = major depressive disorder, OCD = obsessive-compulsive disorder; BDI-II = Beck Depression Inventory-II; WST = Test of Word Power; OCI-R = Obsessive-Compulsive Inventory Revised, ^1^ = based on *n* = 45;

^2^ = based on *n* = 53.

### 2.2 Materials

The Mini International Neuropsychiatric Interview (M.I.N.I.; [33] was used to verify a diagnosis of MDD or OCD and possible comorbidities as well as any psychological disorder in healthy controls (exclusion criteria). To quantify depressive symptoms in the MDD sample, the Hamilton Depression Rating Scale (HDRS; [34]), a semi-structured interview consisting of 17 items, was administered. The HDRS is the most commonly used interview-based measure of depression with well-documented reliability and validity (e.g., [35]). The Yale-Brown Obsessive Compulsive Scale (Y-BOCS; [36]) is a semi-structured interview, was administered to assess severity of obsessions and compulsions. The German version of the Y-BOCS has shown good internal consistency and inter-rater reliability [37]. Next to interviews, questionnaires were used to assess psychopathology and perfectionism. Severity of depressive symptoms was assessed using the German version of the Beck Depression Inventory (BDI-II; [38]). Severity of distress caused by OCD symptoms was measured with the German version of the Obsessive Compulsive Inventory-Revised (OCI-R; [39]). Explicit perfectionism was assessed using the Multidimensional Perfectionism Scale of Frost (FMPS; German version; [40]. It is a 35-item self-report measure with a 5-point Likert scale. It comprises six subscales of which three were analyzed in this study. The subscale of “personal standards” corresponds to the perfectionistic-strivings factor of perfectionism [22]. The factorial structure of the FMPS showed a better fit when the subscales of “concerns over mistakes” and “doubts about actions” are combined into one composite score of “perfectionistic concerns” [40]. The FMPS has shown good reliability and validity. The subscales as well as the total score show satisfactory to high internal consistencies [40]. The test of Word Power was used to estimate verbal intelligence (WST; [41]).

Table 1 displays demographic and psychopathological characteristics of the OCD patients, the MDD patients and the healthy controls. Regarding the disorder specific interviews, patients with MDD showed a total score of *M* = 19.29, *SD* = 7.58 on the HDRS, indicating a moderate level of MDD symptoms [42]. Patients with OCD showed a total score of *M* = 21.55, *SD* = 6.30 on the Y-BOCS, indicating a moderate level of OCD symptoms [43] with *M* = 11.35, *SD* = 3.67 for the obsessions subscale and *M* = 10.20, *SD* = 3.22 for the compulsions subscale. Symptoms of depression ranged between moderate and severe for patients with MDD and light to moderate for patients with OCD as measured with the BDI-II. More than half of both patient groups affirmed a comorbid diagnosis and most patients received pharmacological medication.

### 2.3 Perfectionism single-category IAT

The Single-Category IAT (SC-IAT) measures reaction times to combinations of words. In the perfectionism IAT [32] the categories “achievement oriented” and “me” were paired in the compatible block and “achievement oriented” and “others” was paired in the incompatible block. The SC-IAT is based on the assumption that the reaction times decrease when the two categories assigned to the same key have a stronger association in memory network than the other combination. See Table 2 for an overview of the perfectionism SC-IAT.

**Table 2:** Overview of blocks presented in the perfectionism single category IAT.

Block Number	Block Name	Number of Trails
1	Practice Compatible: me + achievement-oriented vs. others)	24 Trials
2	Test Compatible: me + achievement-oriented vs. others)	72 Trials
3	Practice Incompatible: me vs. others + achievement-oriented	24 Trials
4	Test Incompatible: me vs. others + achievement-oriented	72 Trials

**Fig 1:**
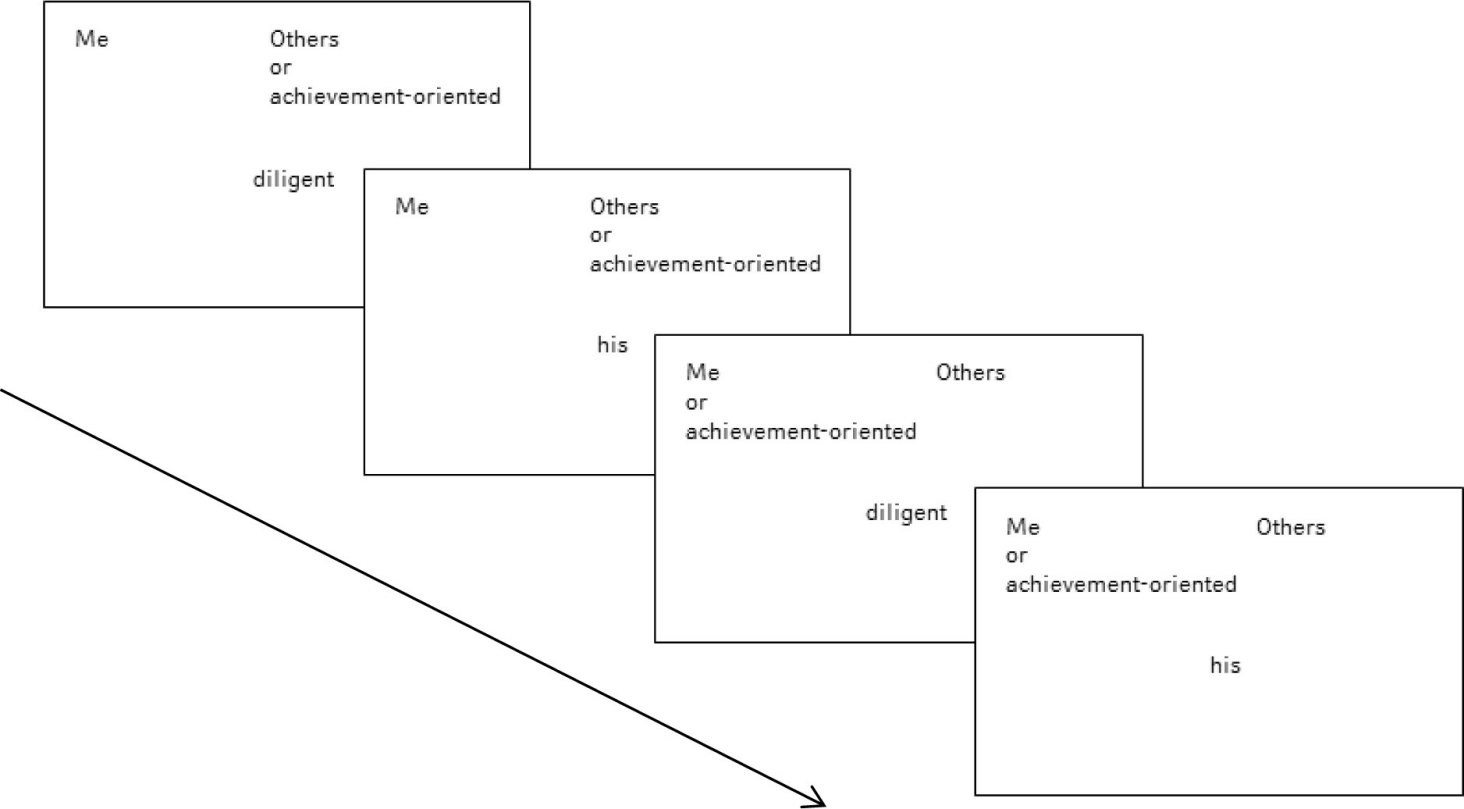
Examples of the four blocks of the perfectionism SC-IAT. In each block the attribute labels were presented and remained on the top left and top right corner of the screen, while the target category was presented on the top left in the one block and on the top right in the other block. The stimuli were displayed in the middle of the screen and had to be classified into the respective category.

See Fig 1 for a schematic presentation of the SC-IAT. Response latencies were the elapsed time between the start of each stimulus presentation and the correct response. Participants were randomly assigned to either start with the combination of me + achievement oriented or of others + achievement oriented. Feedback was given 150 ms after each trial. In case of a correct answer, a green circle appeared and the stimulus disappeared. In case of a wrong answer, a red “X” appeared and participants had to correct their response by pressing the other key. The next stimulus appeared 250 ms after the correct categorization had been made. In case the correct response was not given within 2000 ms, “Please respond more quickly” appeared on the screen for 500 ms and then the next trial started.

The perfectionism SC-IAT was administered using the program Inquisit [44]. A similar SC-IAT showed good construct and predictive validity in a sample of students [32]. To enhance reliability (Cronbach’s α = .54 and α = .74; [32], we planned to include ten instead of four words in the achievement oriented category. To select stimuli, 20 words related to achievement oriented were chosen by the first author. Ratings were performed by 21 German independent who were recruited via a social media platform (33.3% male) with a mean age of *M* = 28.95 (*SD* = 3.95) years and an average of *M* = 18.7 years of education (*SD* = 2.25). The words were rated on Likert-scales according to how well the word fitted to achievement oriented (1 = not at all; 6 = completely), how understandable (1 = not at all; 6 = completely), and specific the word was (1 = not at all, 4 = very). Consequently, ten words were selected according to the following steps: 1.) how precise the word was, was scored as 3 (moderately) or higher, 2.) how understandable the word was, was scored as 4 (well) or higher, 3.) the ten words that were rated to fit best to achievement oriented were chosen. The selected words all fitted well to fully to achievement oriented (range: 4.05–5.57). For an overview of the final stimuli set, see Table 3.

**Table 3:** Target and attribute stimuli used in the perfectionism SC- IAT. German words that were used in the assessment are provided in parentheses.

Attribute Stimuli		Target stimuli
Me	Others	Achievement-oriented
Me (mir)	She (sie)	efficient (tüchtig)
My (mein)	He (er)	competitive (wetteifernd)
Me (mich)	Her (ihr)	unerring (zielsicher)
I (ich)	His (sein)	ambitious (ambitioniert)
Self (selbst)	Others (andere)	striving (strebsam)
		diligent (fleißig)
		determined (entschlossen)
		driven (ehrgeizig)
		persistent (ausdauernd)
		purposeful (zielstrebig)

### 2.4 Procedure

After completing the informed consent, participants filled out the questionnaires. Patients with MDD did not fill out the OCI-R. Subsequently, participants were interviewed to gain socio-demographic data and psychopathological data using the M.I.N.I‥ Afterwards, patients were assessed with the respective disorder-specific interview (Y-BOCS for patients with OCD and HDRS for patients with MDD). Then, participants completed the test on verbal intelligence (WST) and the perfectionism SC-IAT.

### 2.5 Sample size estimation

The necessary amount of participants was calculated to achieve 90% test power at an error rate of α = .05 for the univariate ANOVA using G*Power [45]. The meta-analysis by Limburg and colleagues [14] showed a moderate association between perfectionism and psychopathology (including MDD and OCD) of *d* = 0.54. Using an expected medium effect of *d* = 0.54, 49 participants for each group would be necessary to find a difference between MDD patients, OCD patients, and healthy controls.

### 2.6 Strategy of data analysis

The scoring of the perfectionism SC-IAT followed the improved scoring algorithm as described in Greenwald, Nosek, and Banaji [46]. Only trials from test blocks, in which the response time was between 400 ms and 10,000 ms were used for calculating the *D*_*2*_-score.

$$D2=\frac{Mean\;Response\;Latencies\;(incompatible\;trials)-Mean\;Response\;Latencies\;(compatible\;trials)}{\text{Pooled}\;\text{Standard}\;\text{Deviation}\;(\text{incompatible}\;\text{and}\;\text{compatible}\;\text{trials})}$$

Higher *D*_*2*_-scores indicate faster reactions to me + achievement oriented compared to others + achievement oriented. Thus, higher *D*_*2*_-scores are thought to be interpreted as a measure of an achievement-oriented self-concept in the associative system. We computed a split-half reliability by splitting up the trials of the IAT test blocks into odd and even trials. The result showed acceptable to good split-half reliability, *r* = .77 (after Spearman-Brown correction). To test our hypothesis that patients would show higher scores regarding both factors of perfectionism using self-report measures. We planned to compute a 3 Groups (MDD, OCD, Healthy) x 2 self-report scores (perfectionistic strivings, perfectionistic concerns) MANOVA. To test our hypothesis that both groups of patients with should higher scores regarding the perfectionistic-strivings factor when using the SC-IAT, we planned to calculate a univariate ANOVA for the SC-IAT with perfectionism as the dependent variable and groups (MDD, OCD, healthy) as independent variable. We ran the same analyses in patients with OCD who did not report a comorbid MDD or comorbid dysthymia (*n* = 30) and patients with MDD who did not report a comorbid OCD (*n* = 48). To complement analyses and to test whether the two patient groups were similar regarding the perfectionism scores, we planned to also compute Bayesian ANOVAs to clarify the intensity of the evidence that the data provide for an alternative versus null hypothesis (e.g., [47]). Namely, Bayes Factors (th10) were calculated to confirm possible differences between the groups and to assess the similarities across groups regarding perfectionism as measured by self-report or the SC-IAT. One of the advantages of a BF analysis is that it allows for a direct test on the relative evidence of a hypothesis over another. It quantifies how much more likely the data is under a hypothesis (e.g., three groups differ) than under another hypothesis (e.g., no group differences; [48]. Thus, BF gives a continuous measure, which goes beyond the dichotomous judgement of null-hypothesis testing. We used JASP (Version 0.9) to compute BFs [49]. JASP assigns Cauchy priors to effect sizes and estimates two models: the null model, assuming no group differences (i.e., having only the grand mean of an outcome, such as the FMPS score); the alternative model, assuming the effect of the groups (given as distance to the grand mean). BF10 was then defined as the relative likelihood of these two models with a value of > 1 indicating support in favor of the alternative model. Pearson-product correlations were computed to test the relationship between the different measures of perfectionism within each group.

## 3. Results

### 3.1 Demographic and psychopathological scores

No differences between the three groups emerged for any of the demographic characteristics (see Table 1). As expected, psychopathological scores were higher in patients compared to healthy controls. Both groups of patients reported significantly higher depressive symptoms as indicated by the BDI-II total scores compared to the healthy control group (MDD: *t*(117) = 14.00, *p* < .001, *d* = 2.60; OCD: *t*(115) = 10.21, *p* < .001, *d* = 1.91) and patients with MDD scored higher than patients with OCD, *t*(106) = 4.17, *p* < .001, *d* = 0.81. The groups did not differ regarding comorbid disorders or psychotropic medication. According to the M.I.N.I seven patients with MDD also fulfilled criteria for comorbid OCD, 22 patients with OCD fulfilled criteria for MDD and/or dysthymia (of those 22 patients, five patients fulfilled the criteria for both MDD and dysthymia).

### 3.2 Self-report data on perfectionism

See Table 4 for an overview of data on perfectionism using self-report measures and the SC-IAT. The MANOVA testing differences regarding self-reported perfectionism showed that the three groups differed significantly, *F*(4, 322) = 16.67, *p* < .001, η_p_^2^ = .17. Including gender as a covariate into the analysis did not change the results. Follow-up tests using univariate ANOVAs revealed that the three groups differed significantly regarding both the perfectionistic-strivings factor, *F*(2,164) = 9.06, *p* < .001, η_p_^2^ = .10, and the perfectionistic-concerns factor, *F*(2,164) = 35.64, *p* < .001, η_p_^2^ = .31. When compared to the healthy control group, patients with MDD and patients with OCD scored higher on the perfectionistic-strivings factor (MDD: *t*(115) = 3.39, *p* < .001, *d* = 0.14; OCD: *t*(107) = 3.73, *p* < .001, *d* = 0.73) with small to medium effects and the perfectionistic-concerns factor (MDD: *t*(115) = 5.49, *p* < .001, *d* = 1.02; OCD: *t*(107) = 8.79, *p* < .001, *d* = 1.72) with large effects. Whereas patients with MDD and OCD did not differ with regard to the perfectionistic-strivings factor (*t*(100) = 0.34, *p* = .73, *d* = 0.07), patients with OCD scored higher on the perfectionistic-concerns factor compared to patients with MDD (*t*(100) = 2.63, *p* = .01, *d* = 0.53) with a medium effect. The same analyses in patients with OCD with no comorbid MDD and no comorbid dysthymia and patients with MDD with no comorbid OCD revealed similar results. The only difference to the results reported above was that patients with OCD and patients with MDD did not differ regarding perfectionistic-concerns factor (*t*(76) = 1.72, *p* = .09, *d* = 0.40).

**Table 4:** Perfectionism data: Mean (standard deviation).

	MDD patients (*n* = 55)	OCD patients (*n* = 55)	Healthy controls (*n* = 64)
*Self-report Measure (FMPS)*			
Perfectionistic Strivings (FMPS-PS)	23.18 (5.79)	23.55 (5.11)	19.42 (6.17)
Perfectionistic Concerns (FMPS-CMD)	37.24 (12.33)	43.26 (10.54)	25.95 (9.89)
*Indirect Measure (SC-IAT)*			
*D*_*2*_-Score	-.05 (.25)	- .11 (.29)^1^	-.09 (.34)^2^
Errors (%)	5.75	6.12	5.82

*Note*: FMPS = Multidimensional Perfectionism Scale of Frost, FMPS-PS = Personal standard subscale, FMPS-CMD = Subscales of Concerns over Mistakes and Doubts about Actions, SC-IAT = Single Category IAT, ^1^ = based on *n* = 46;

^2^ = based on *n* = 60.

When using Bayesian ANOVAs, strong evidence in favor of the alternative over the null hypothesis (BF10 > 131.74) for the two factors was found. Post-hoc tests with posterior odds (i.e., BFs corrected for multiple tests) suggest that it is more likely that each patient group differs from the healthy controls (i.e., the alternative hypotheses) than that they do not differ (i.e., the null hypotheses; posterior odds > 17.6). The comparisons between MDD and OCD revealed that it is more likely that the two patient groups do not differ regarding the perfectionistic-strivings factor (i.e., null hypothesis: MDD = OCD) than that they differ (i.e., alternative hypothesis: OCD ≠ MDD; posterior odds = 0.13). Regarding the perfectionistic-concerns factor, strong evidence was found in favor of the alternative hypotheses for the patient-control comparisons (posterior odds > 30134.26). When comparing MDD and OCD, the data provide small evidence in favor of the alternative hypothesis for the MDD-OCD comparison (posterior odds = 2.5), suggesting that the two patient groups differ regarding the perfectionistic-concerns factor. As the perfectionistic-concerns factor comprises both the concern over mistakes and the doubts about actions subscale, we calculated an exploratory Bayesian ANOVA to test whether the two patient groups also differed only regarding the concern over mistakes scale. Anecdotal evidence in favor of the null hypothesis was found (BF10 = 0.86; posterior odds = 0.51), suggesting that the groups may not differ regarding the concern over mistakes scale.

### 3.3 Indirect data on perfectionism

On the SC-IAT, the three groups did not differ in number of error trials, *F*(1, 161) = 0.07, *p* = .93, η_p_^2^ = .001. Including gender as a covariate into the analysis did not change the results. The means and standard deviations of the perfectionism SC-IAT scores are shown in Table 4. Notably, the *D*_*2*_-scores did not differ from 0, which can be interpreted as neither a tendency of perfectionistic strivings for others nor for oneself [50]. Contrary to our hypothesis, the three groups did not differ in their perfectionistic self-concepts according to the SC-IAT, *F*(1, 161) = 0.48, *p* = .62, η_p_^2^ = .006. The same analyses in patients with OCD with no comorbid MDD and no comorbid dysthymia and patients with MDD with no comorbid OCD revealed similar results. Similarly, using the Bayes Factor, the data provide strong evidence in favor of the null hypothesis (i.e., MDD = OCD = controls) with BF10 of 0.099, suggesting that the three groups do not differ.

### 3.4 Correlational analyses

In all groups the subscales for perfectionistic strivings and perfectionistic concerns of the FMPS significantly correlated with each other (OCD: *r* = .56, *p* < .001; MDD: *r* = .50, *p* < .001, healthy controls: *r* = .61, *p* < .001). No significant correlations emerged between the perfectionism score on the SC-IAT and either factor of the FMPS for patients with OCD, *r*s < .30, *p*s > .07, or healthy controls, *r*s < .03, *p*s > .57. For patients with MDD, no significant correlations were found between the perfectionistic-concerns factor and perfectionism scores on the SC-IAT, *r* = .02, *p* = .88. However, the perfectionistic-strivings factor as measured by the FMPS correlated significantly with the perfectionism score on the SC-IAT, *r* = .33, *p* = .02, in patients with MDD.

## 4. Discussion

To the best of our knowledge, this study is the first to use Bayesian statistics to assess the similarity between patients with MDD and OCD regarding self-reported perfectionism. Furthermore, it is the first to complement a self-report measure (FMPS) of perfectionism with and an indirect measure (SC-IAT) in patients with different psychological disorders. However, the indirect measure only assesses perfectionistic strivings, not perfectionistic concerns.

### 4.1 Self-report data on perfectionism

In replication of previous results [14], patients with MDD and patients with OCD reported significantly higher perfectionism scores on both the perfectionistic-strivings factor and the perfectionistic-concerns factor of a self-report measure, when compared to the healthy control group. Our results regarding higher perfectionistic strivings in the two patient groups compared to the healthy controls further call into question the notion that perfectionistic strivings could be seen as healthy or adaptive [51, 52]. Indeed, previous research on psychopathology has also shown that perfectionistic strivings are elevated across various disorders [14]. Theoretical conceptualizations suggest that it is important to differentiate between the pursuit of excellence, which is assumed to be adaptive and the pursuit of perfectionism, which is assumed to be maladaptive. Whereas the pursuit for excellence has been conceptualized as setting high standards, the pursuit of perfectionism refers to exceedingly high aims and striving intensity, with goals that can never be reached [12]. Future studies in clinical samples could test whether the pursuit of excellence and pursuit of perfectionism are differentially associated with psychopathology and other maladaptive outcomes.

Using Bayesian statistics, we were also able to assess whether the two patient groups reported similar results regarding perfectionism. As expected, the two groups of patients did not differ in their self-reported scores regarding the perfectionistic-strivings factor. However, contrary to our expectations, patients with OCD reported higher scores on the perfectionistic-concerns factor, but only when the subscale “doubts about actions” of the FMPS was included. The perfectionistic-concerns factor includes the two subscales of the FMPS “concerns over mistakes” and “doubts about actions” (according to factor analysis see [40]). The scale “doubts about actions”, however, comprises items that were taken from a measure of OCD symptoms (Maudsley Obsessive-Compulsive Inventory; [53]). Exploratory Bayesian analysis showed some evidence that the concern over mistakes subscale (without the doubts about actions scale) did not differ between the two patient groups.

The similarity between the two disorders gives some support to the transdiagnostic perspective which argues that similar processes are apparent across disorders [4]. Based on our results one of those processes could be perfectionism, with both the perfectionistic strivings and perfectionistic concerns factor. However, our results undermine the importance of how careful researchers should be when selecting and evaluating items to assess possible transdiagnostic processes. Due to the overlap of items assessing perfectionistic concerns with a scale assessing symptoms of OCD, the perfectionistic-concerns factor is not fully comparable between OCD patients and patients with other disorders. This may lead to an overestimation of perfectionistic concerns in individuals with OCD, which would ultimately be a problem when offering transdiagnostic treatments for perfectionism [54].

### 4.2 Indirect data on perfectionism

In line with our hypothesis, the two groups of patients did not differ regarding the perfectionistic-strivings factor when measured with the SC-IAT. However, contrary to our hypothesis, the patients also showed similar reaction times as healthy controls. This results stands in contrast to cognitive models of MDD and OCD that posit dysfunctional beliefs to be especially apparent in the associative system [6, 7, 25]. As stated above, the perfectionistic-strivings factor of perfectionism has also been acknowledged as a possibly adaptive form of perfectionism [13]. Accordingly, this factor leads to distress if individuals continue to strive for those goals even when they are not attainable [55]–in contrast to the pursuit of excellence [49]. Thus, the flexibility to adjust one’s standards and consequently pursue or relinquish goals could be regarded as a coping skill. According to the assumptions by Beck and Alford [9] in a depressive phase dysfunctional beliefs are more profound, while coping skills are suppressed. Thus, it is possible that perfectionistic concerns are the dominant dysfunctional belief activated in the associative system. Higher scores on self-report measures regarding the perfectionistic-strivings factor may be due to a reduced flexibly to adapt one’s own personal standards [55]. This would mean that the two factors are closely related to each other. This assumption is supported by the results of our correlational analyses. Similar results were found in a meta-analysis, which included both disorders [14].Future studies are necessary to test the perfectionistic-concerns factor in the associative system using indirect measures. However, to the best of our knowledge, this is the first study that uses an indirect measure to assess perfectionism in patient samples that reports psychometric properties. The SC-IAT was chosen because its predictive validity was good [32]. According to the stimulus rating, the current German stimuli fit well with the concept “achievement-oriented” and were easily understandable. The SC-IAT in the current study showed an acceptable to good split-half reliability, which is slightly better compared to the validation study [32] and higher than most indirect measures [30]. Indirect measures may show a great potential in assessing processes underlying disorders. This may especially important when trying to reliably assess transdiagnostic processes in individuals in order to offer the right form of therapy. However, when creating or using indirect measures efforts have to be undertaken to assess, report, and ultimately increase reliability and validity [28].

### 4.3 Limitations

This study has a number of limitations. The SC-IAT only assessed the perfectionistic-strivings factor of perfectionism. As stated above a reliable and valid indirect measure should be developed to assess the perfectionistic-concerns factor. Second, the groups were comparable regarding the number of patients with comorbid disorders. Yet, about a third of the patients with OCD also showed symptoms of MDD or dysthymia and seven patients with MDD reported symptoms of OCD. This symptom overlap restricts the internal validity of the study as well as disorder-specific interpretations of the results. However, it further support transdiagnostic efforts to assess vulnerabilities such as dysfunctional beliefs across the two disorders as the comorbidity between the two disorders is high [2, 3]. The results would also have profited from including the OCI-R in the MDD group to be able to more specifically assess symptoms of OCD in this group and compare the level of OCD symptoms with that of the patients with OCD. Third, our sample size estimation was conducted to test whether the clinical groups showed higher scores compared to the healthy controls. However, since we were also interested in calculating differences between the two patient groups separately (MDD vs. healthy controls and OCD vs. healthy controls) we should have conducted our sample size estimation based on these analyses. However, since the effect found in the current study was larger compared to the expected effect, we still found group differences in the t-tests. Fourth, the factor perfectionistic concerns of the FMPS shows a substantive, nontrivial overlap with symptoms of OCD as the doubts about actions subscale includes items taken from the Maudsley Obsessive-Compulsive Inventory [53]. Thus, it may not be surprising that patients with OCD only reported higher perfectionistic concerns compared to patients with MDD, when the doubts about actions scale was included. Fifth, associative studies are an important first step to understand whether a process is apparent across disorders and may be considered a transdiagnostic process. However, Harvey and colleagues [4] argued that a process is defined as an aspect of cognition or behavior that contributes to the *maintenance* of a disorder. Recent meta-analyses showed that both perfectionistic concerns and perfectionistic strivings may serve as risk factors to increase anxiety [56] and depressive symptoms [15]. Moreover, some evidence exists that perfectionism may serve as a maintaining factor across disorders [57]. To extend these findings, future studies should assess perfectionism in longitudinal studies and in experimental studies in which perfectionism is induced to test its effect on psychological symptoms.

### 4.4 Clinical implications

A transdiagnostic perspective to diagnose and treat psychological problems has become increasingly popular [4, 58, 59]. It gives the opportunity to specifically target underlying processes, which may enhance treatment effects. Furthermore, it considers the heterogeneity of disorders and the high comorbidity rates. Our results give further evidence, that perfectionistic concerns may be a transdiagnostic process which is similarly associated with MDD and OCD. Therefore, it may be helpful for those patients to receive specific treatments that focus on perfectionism (e.g., [54]). One challenge in offering the appropriate treatment that targets a specific transdiagnostic process to a patient is the assessment of that process. Our results showed that self-report measures do not fully overlap with our indirect measure of perfectionism, even though both the associative and reflective system have been proposed to be relevant for dysfunctional beliefs. Furthermore, our results revealed that even the self-report measure shows some disadvantages and may overestimate perfectionistic concerns in patients with OCD. Future studies are necessary to further evaluated diagnostic tools to assess perfectionism as a transdiagnostic process.

## 5. Conclusion

To the best of our knowledge, this is the first study that complements self-report measures with an indirect measure of perfectionism in patients with different psychopathologies and directly assesses whether the two disorders, namely MDD and OCD, show similar perfectionism scores. In replication of previous findings, both the perfectionistic-strivings factor and the perfectionistic-concerns factor were elevated more strongly in patients with MDD and OCD compared to a healthy control group when assessed using self-report measures. Furthermore, the two groups did not differ regarding perfectionistic strivings. Patients with OCD only reported higher perfectionistic-concerns scores, when perfectionistic concerns scores included the “concerns over mistakes” as well as the “doubts about actions” subscale of the FMPS. However, the “doubts about actions” subscale shows a strong overlap with a measures of OCD symptoms. When only the “concerns over mistakes” subscale was considered, the patients with MDD and OCD did not differ regarding perfectionistic concerns. The indirect measure of perfectionism has given first evidence that the perfectionistic-strivings factor of perfectionism is not more pronounced in patients with MDD and OCD compared to healthy controls in the associative system. These results may suggest that contrary to cognitive models of the two disorders the perfectionistic-strivings factor of perfectionism is not as important. More research is necessary, including indirect measures to assess the perfectionistic-concerns factor of perfectionism in the associative system.

## Supporting information

S1 File (SAV)
